# Balloon pulmonary angioplasty followed by pulmonary endarterectomy: Combination treatment for high-surgical-risk patients with chronic thromboembolic pulmonary hypertension

**DOI:** 10.1093/icvts/ivad031

**Published:** 2023-02-24

**Authors:** Yusuke Shimahara, Shun Suzuki, Toshiki Fujiyoshi, Sayaka Honda, Nobusato Koizumi, Jun Yamashita, Yuichi Sasaki, Ryosuke Ito, Lisa Takahashi, Michikazu Nakai, Hitoshi Ogino

**Affiliations:** Department of Cardiovascular Surgery, Tokyo Medical University, Tokyo, Japan; Department of Cardiovascular Surgery, Tokyo Medical University, Tokyo, Japan; Department of Cardiovascular Surgery, Tokyo Medical University, Tokyo, Japan; Department of Cardiovascular Surgery, Tokyo Medical University, Tokyo, Japan; Department of Cardiovascular Surgery, Tokyo Medical University, Tokyo, Japan; Department of Cardiology, Tokyo Medical University, Tokyo, Japan; Department of Cardiology, Tokyo Medical University, Tokyo, Japan; Department of Cardiology, Tokyo Medical University, Tokyo, Japan; Department of Cardiology, Tokyo Medical University, Tokyo, Japan; Department of medical and health information management, National Cerebral and Cardiovascular Center, Osaka, Japan; Department of Cardiovascular Surgery, Tokyo Medical University, Tokyo, Japan

**Keywords:** Combination therapy, Preceding balloon pulmonary angioplasty, Pulmonary endarterectomy, Chronic thromboembolic pulmonary hypertension

## Abstract

**OBJECTIVES:**

Our goal was to evaluate the combined effects of balloon pulmonary angioplasty (BPA) followed by pulmonary endarterectomy (PEA) to treat high-surgical-risk patients with chronic thromboembolic pulmonary hypertension (CTEPH).

**METHODS:**

This study included 58 patients with CTEPH who had pulmonary vascular resistance of ≥1000 dyn·s/cm^5^, mean pulmonary arterial pressure (mPAP) of ≥45 mmHg or mPAP of 38–44 mmHg with comorbidities. Of these, 21 patients underwent the combined therapy of BPA followed by PEA (BPA group) and 37 underwent direct PEA (non-BPA group). Preoperative and postoperative results were compared between the 2 groups. An early postoperative composite event comprised the postoperative use of extracorporeal membrane oxygenation or intra-aortic balloon pump, in-hospital death, rescue BPA, prolonged ventilation, tracheostomy, prolonged stay in the intensive care unit, deep sternal wound infection and cerebral infarction.

**RESULTS:**

Before the first intervention (before BPA or direct PEA), patients in the BPA group had a higher mPAP than those in the non-BPA group. After undergoing BPA before PEA, the BPA group demonstrated significantly decreased mPAP and pulmonary vascular resistance (43 vs 52 mmHg, *P* < 0.001; 636 vs 965 dyn·s/cm^5^, *P* = 0.003, respectively) and significantly increased cardiac output (4.1 vs 3.5 l/min, *P* = 0.041). Notably, the number of patients with the early postoperative composite event was significantly lower in the BPA group than in the non-BPA group (4.8% vs 35.1%, *P* = 0.011).

**CONCLUSIONS:**

Compared with direct PEA, the combination therapy of BPA followed by PEA can be a feasible and effective risk-reduction strategy for high-surgical-risk patients with CTEPH.

**Figure:**
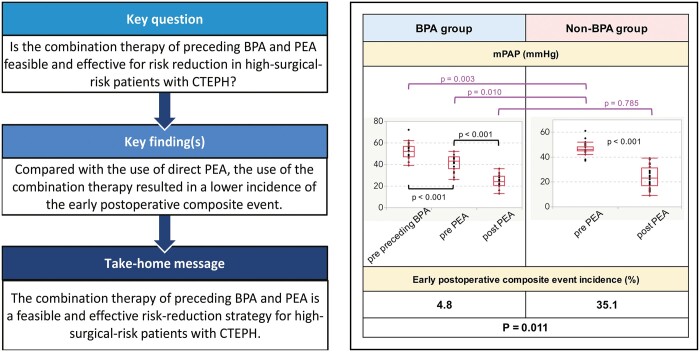

## INTRODUCTION

Pulmonary endarterectomy (PEA) is considered the first-line therapy in the treatment algorithm for chronic thromboembolic pulmonary hypertension (CTEPH) [1, 2]. However, both pulmonary arterial hypertension (PAH)-targeted medical treatment (MT) and balloon pulmonary angioplasty (BPA) are recommended in the algorithm [2,3]. Notably, identification of the morphology of the pulmonary arterial (PA) lesion is crucial for the management of patients with CTEPH because whether PEA, BPA, or MT should be performed depends on the location of the PA lesions: PEA for the main, lobular and proximal segmental branches; BPA for the distal segmental and subsegmental branches; and MT for the more distal subsegmental branches. In contrast, several recently published reports on the use of combination therapy have recommended MT for patients with residual PAH after PEA (4), whereas the combination MT and BPA has been reported to be effective in improving the survival of patients considered to be inoperable (5). Moreover, several researchers have reported that an additional BPA following PEA is safe and effective for patients with residual PAH (6, 7). However, the efficacy of receiving nonsurgical therapies before PEA is unknown; in particular, there is little information about the effect of performing BPA before PEA. Thus, the goal of the present study was to evaluate the postoperative outcomes following the combination therapy of BPA followed by PEA for high-surgical-risk patients with CTEPH.

## METHODS

### Ethical statement

This retrospective observational study was conducted at Tokyo Medical University. The institutional ethics board approved this study (Tokyo Medical University: T2022-0014; 10 May 2022), and the requirement for obtaining informed consent from the patients was waived because of the retrospective nature of the study.

### Study design

**Figure 1:**
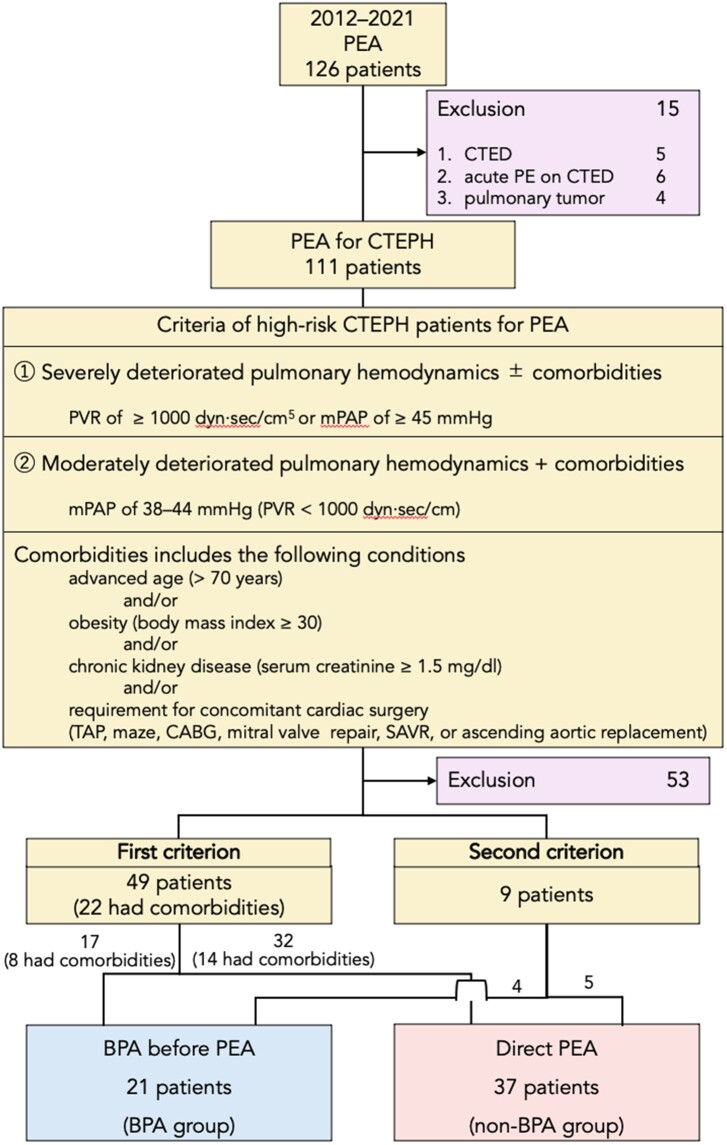
Inclusion and exclusion criteria for patient selection. BPA: balloon pulmonary angioplasty; CABG: coronary artery bypass grafting; CTED: chronic thromboembolic disease; CTEPH: chronic thromboembolic pulmonary hypertension; mPAP: mean pulmonary arterial pressure; PE: pulmonary embolism; PEA: pulmonary endarterectomy; PVR: pulmonary vascular resistance; SAVR: surgical aortic valve replacement; TAP: tricuspid annuloplasty.

Patients with CTEPH who underwent PEA between 2012 and 2021 were enrolled in the present study (Fig. 1). Among them, patients with severe pulmonary hypertension with preprocedural pulmonary vascular resistance (PVR) of ≥1000 dyn·s/cm^5^ or an mPAP of ≥45 mmHg, which is the first criterion to identify patients at high surgical risk, were included (*n* = 49). In addition, as per the second criterion (*n* = 9), patients with moderate pulmonary arterial hypertension with an mPAP of 38–44 mmHg were considered to be at a high risk for surgery if they had comorbidities, such as age >70 years, body mass index ≥30 kg/m^2^ and a serum creatinine level of ≥1.5 mg/dl, or if they required concomitant cardiac operations, such as tricuspid annuloplasty, coronary artery bypass grafting, a maze procedure, surgical aortic valve replacement, mitral valve repair or ascending aortic replacement. Of the patients who met the first or second criterion, those who underwent BPA before PEA were included in the BPA group (*n* = 21), whereas those who underwent direct PEA were included in the non-BPA group (*n* = 37). Although these high-surgical-risk patients were considered candidates for having BPA before PEA, a decision on whether to perform the BPA was made by our CTEPH team based on the patient’s condition and surgical schedules. In the BPA group, 12 (57.1%) patients underwent BPA before PEA at Tokyo Medical University, whereas the remaining 9 patients underwent BPA before PEA at other hospitals and were referred to our hospital for the treatment of residual PAH. The number of patients in the BPA group has increased noticeably since 2017 (Supplementary Table S1).

Preoperative patient characteristics, data on having BPA before PEA, changes in data on right heart catheterization, early postoperative outcomes and survival after PEA were evaluated in this study. An early postoperative composite event involved at least 1 of the following complications: use of postoperative extracorporeal membrane oxygenation (ECMO), use of postoperative intra-aortic balloon pump (IABP), in-hospital death, rescue BPA, prolonged ventilation (>3 days), tracheostomy, deep sternal wound infection, cerebral infarction and prolonged stay in the intensive care unit (>10 days). Rescue BPA was required to wean patients with severe respiratory dysfunction caused by residual PAH off mechanical circulatory or ventilatory support. Cerebral infarction was defined as the condition of at least being unable to perform all previous activities due to the disruption of the blood supply to the brain.

Clinical follow-up data were obtained from hospital clinical records of the Tokyo Medical University from May 2022 to July 2022. Telephone- or questionnaire-based follow-ups were conducted for patients who did not visit our centre after 2021.

### Balloon pulmonary angioplasty procedure preceding a pulmonary endarterectomy

The main targets of the PA lesions were the right ascending segmental, middle lobe, left lower segmental and subsegmental arteries, which were difficult to access during PEA. However, as a risk-reduction procedure, surgically accessible arteries were also targeted to improve pulmonary haemodynamics. A soft-tipped 6-Fr guiding catheter was inserted into the right or left PA via a short 9-Fr sheath from the femoral vein to perform selective pulmonary angiography. After crossing the wire across the targeted lesion, a monorail balloon catheter of appropriate diameter was used to dilate the lesion. In patients exhibiting signs of vascular injury or reperfusion pulmonary oedema, noninvasive positive pressure ventilation (NPPV) was used to prevent the deterioration of these complications [7].

### Pulmonary endarterectomy procedure and perioperative management

PEA was performed using techniques similar to those established by the San Diego group [8, 9]; it was performed with cycles of 15 min of deep hypothermic circulatory arrest followed by 10 min of systemic reperfusion at a core temperature of 18°C. The surgical classification of pulmonary occlusive disease related to thromboembolic materials was based on the San Diego group classification [9]. Venoarterial ECMO was initiated as a bridge to recovery using peripheral or central access when weaning from cardiopulmonary bypass was unsuccessful because of respiratory and/or cardiac failure [10]. Furthermore, IABP was initiated intraoperatively when hypotension persisted.

Intravenous MT (epoprostenol) was commonly used preoperatively to improve pulmonary haemodynamics in such high-surgical-risk patients before 2014. In addition, oral MT (mainly riociguat) was widely used preoperatively throughout the study period. The administration of heparin was initiated the next day after PEA and continued until oral warfarin was resumed with a target international normalized ratio of 2.0–3.0. Early right heart catheterization after PEA was performed approximately 3 weeks after the operation.

### Statistical analyses

Continuous variables, compared using the Mann–Whitney U test, are expressed as medians with interquartile range. Categorical variables, compared using the Pearson χ^2^ or the Fisher exact test, are expressed as frequency (percent). Odds ratios and 95% confidence intervals for assessing the factors associated with the composite event were calculated using univariate logistic regression models. Covariates that potentially affected the composite event and in which the total number of patients in each covariate was ≥5 were selected from Tables 1 and 2. Multivariate logistic regression analysis was performed using covariates that were found to be significantly associated with the composite event based on the results of the univariate analysis. A *P*-value of <0.05 was considered statistically significant. The Kaplan–Meier method (log-rank test) was used to calculate survival between the 2 groups. JMP 16 software (SAS Institute Inc, Cary, NC) and STATA version 16.1 (Stata Corp, College Station, TX) were used for statistical analyses.

**Table 1:** Preoperative patient characteristics

Variable	BPA group	non-BPA group	*P*-value
n	21	37	
Age, years	59 [52.0, 71.5]	63 [52.0, 72.0]	0.710
Females, % (n)	61.9 (13)	64.9 (24)	0.822
Body mass index ≥30 kg/m^2^, % (n)	23.8 (5)	16.2 (6)	0.478
Hypertension	28.6 (6)	48.7 (18)	0.136
Dyslipidaemia	23.8 (5)	13.5 (5)	0.319
Serum creatinine ≥1.5 mg/dl, % (n)	4.8 (1)	5.4 (2)	1.000
Haemodialysis	0	2.7 (1)	1.000
Haemoglobin A1c ≥7.0%, % (n)	4.8 (1)	0	0.362
Atrial fibrillation, % (n)	0	5.4 (2)	0.530
Peripheral vascular disease, % (n)	0	5.4 (2)	0.530
Depression or schizophrenia, % (n)	4.8 (1)	13.5 (5)	0.293
History of cerebral infarction, % (n)	4.8 (1)	10.8 (4)	0.430
Steroid use	0	2.7 (1)	1.000
Thrombotic predisposition, % (n)	23.8 (5)	16.2 (6)	0.478
Current smoker, % (n)	19.1 (4)	8.1 (3)	0.241
Former smoker, % (n)	23.8 (5)	29.7 (11)	0.628
Inotropic agent, % (n)	14.3 (3)	2.7 (1)	0.130
NYHA/WHO functional class			
II, % (n)	38.1 (8)	27.0 (10)	0.382
III, % (n)	47.6 (10)	67.6 (25)	0.252
IV, % (n)	14.3 (3)	5.4 (2)	0.341
Supportive therapy			
Oral PAH-targeted MT	95.2 (20)	83.8 (31)	0.198
Riociguat, % (n)	85.7 (18)	62.2 (23)	0.058
Intravenous PAH-targeted MT			
Epoprostenol	0	24.3 (9)	0.020
Home oxygen therapy, % (n)	85.7 (18)	78.4 (29)	0.493

Data are presented as medians [interquartile range].

BPA: balloon pulmonary angioplasty; MT: medical treatment; NYHA/WHO: New York Heart Association/World Health Organization; PAH: pulmonary arterial hypertension.

**Table 2:** Data on balloon pulmonary angioplasty before pulmonary endarterectomy

Variable	BPA group
Number of sessions/patient	2 [1, 2]
1, % (n)	42.9 (9)
2, % (n)	42.9 (9)
3, % (n)	4.8 (1)
≥4, % (n)	9.5 (2)
Targeted pulmonary artery	
Right lung only, % (n)	14.3 (3)
Left lung only, % (n)	23.8 (5)
Both lungs, % (n)	61.9 (13)
Distribution of targeted pulmonary artery	
Right lung	
Upper lobe, % (n)	57.1 (12)
Middle lobe, % (n)	52.4 (11)
Lower lobe, % (n)	38.1 (8)
Left lung	
Upper lobe except lingular segments, % (n)	28.6 (6)
Lingular segment, % (n)	66.7 (14)
Lower lobe, % (n)	76.2 (16)
Preprocedural inotropic agent, % (n)	23.8 (5)
Complications, % (n)	19.1 (4)
Vascular injury or reperfusion oedema, n	4
Interval from last BPA to PEA, days	44 [22, 134]

Data are presented as medians [interquartile range].

BPA: balloon pulmonary angioplasty; PEA: pulmonary endarterectomy.

## RESULTS

Table 1 shows the preoperative patient characteristics. The median age of the participants was 59 and 63 years in the BPA and the non-BPA groups, respectively. The proportion of patients who received oral MT was high in both groups (95.2% and 83.8% in the BPA and the non-BPA groups, respectively).

###  Balloon pulmonary angioplasty before pulmonary endarterectomy

**Figure 2:**
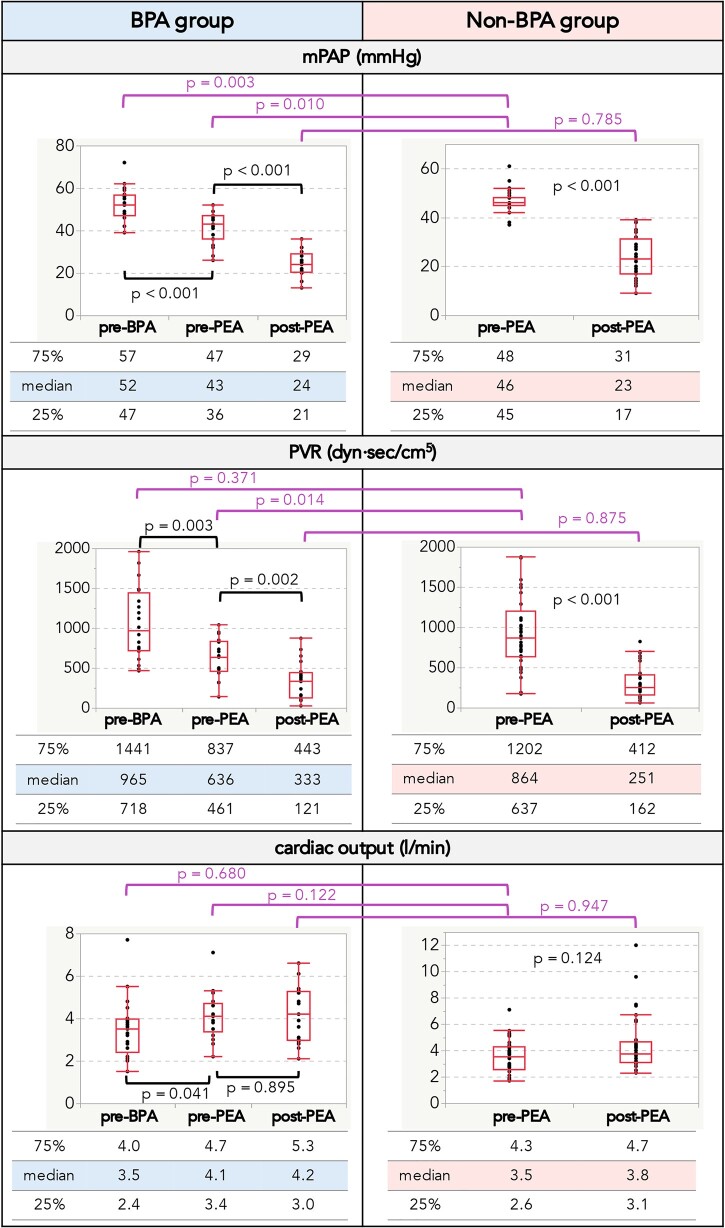
Right heart catheterization data. Data are presented as medians with interquartile ranges. BPA: balloon pulmonary angioplasty; mPAP: mean pulmonary arterial pressure; PEA: pulmonary endarterectomy; PVR: pulmonary vascular resistance

Table 2 shows data from patients who had the BPA before the PEA. Each patient underwent a median of 2 sessions, and 61.9% of patients underwent BPA for both lungs. Four patients (19.1%) experienced complications that improved with the use of NPPV. The median interval from the last BPA to PEA was 44 days. In patients who had the BPA before the PEA, the mPAP and PVR values were found to be significantly decreased (43 vs 52 mmHg, *P* < 0.001; 636 vs 965 dyn·s/cm^5^, *P* = 0.003, respectively), whereas cardiac output was found to be significantly increased (4.1 vs 3.5 l/min, *P* = 0.041) (Fig. 2). Although the patients in the BPA group had higher baseline mPAP values than those in the non-BPA group, preoperative mPAP values were lower in patients in the BPA group.

### Surgical results

Table 3 presents the surgical data. The median cardiopulmonary bypass, aortic cross-clamping and total deep hypothermic circulatory arrest times were similar between the 2 groups. PEA was applied for most lesions that were first treated by BPA. No case of technical difficulty caused by a destroyed PA plane after receiving BPA as a first treatment was observed. The proportion of the patients requiring mechanical support (ECMO and/or IABP) in the BPA group was lower than that in the non-BPA group (4.8% vs 16.2%), but this difference was not statistically significant (*P* = 0.403).

**Table 3:** Surgical data

Variable	BPA group	non-BPA group	*P*-value
Total CPB time, min	218 [205, 265]	253 [209, 296]	0.356
Total aortic cross-clamp time, min	129 [93, 154]	130 [102, 176]	0.241
Total DHCA time, min	38 [32, 56]	48 [35, 63]	0.150
CTEPH surgical classification			
Right lung			
I, % (n)	52.4 (11)	62.2 (23)	0.467
II, % (n)	42.9 (9)	21.6 (8)	0.088
III, % (n)	4.8 (1)	16.2 (6)	0.403
Left lung			
I, % (n)	23.8 (5)	8.1 (3)	0.124
II, % (n)	42.9 (9)	43.2 (16)	0.977
III, % (n)	28.6 (6)	43.2 (16)	0.268
IV, % (n)	4.8 (1)	5.4 (2)	1.000
Proportion of PEA-treated lesions among preceding BPA-treated lesions			
Right lung			
Upper lobe, %	83.3 (10/12)		
Middle lobe, %	90.9 (10/11)		
Lower lobe, %	100 (8/8)		
Left lung			
Upper lobe except lingular segments, %	50.0 (3/6)		
Lingular segments, %	78.6 (11/14)		
Lower lobe, %	81.3 (13/16)		
Concomitant surgery, % (n)	14.3 (3)	29.7 (11)	0.220
TAP, % (n)	0	2.7 (1)	1.000
Maze procedure, % (n)	0	5.4 (2)	0.530
TAP + maze, % (n)	4.8 (1)	5.4 (2)	1.000
Coronary artery bypass grafting, % (n)	9.5 (2)	8.1 (3)	1.000
Mitral valve repair + TAP + maze, % (n)	0	2.7 (1)	1.000
Aortic valve replacement, % (n)	0	5.4 (2)	0.530
Ascending aortic replacement, % (n)	0	2.7 (1)	1.000
Mechanical support requirement after PEA, % (n)	4.8 (1)	16.2 (6)	0.403
ECMO, % (n)	4.8 (1)	8.1 (3)	1.000
Peripheral access, % (n)	0	5.4 (2)	0.530
Central access, % (n)	4.8 (1)	2.7 (1)	1.000
IABP, % (n)	4.8 (1)	16.2 (6)	0.403

Data are presented as medians [interquartile range].

BPA: balloon pulmonary angioplasty; CPB: cardiopulmonary bypass; CTEPH: chronic thromboembolic pulmonary hypertension; DHCA: deep hypothermic circulatory arrest; ECMO: extracorporeal membrane oxygenation; IABP: intra-aortic balloon pump; PEA: pulmonary endarterectomy; TAP: tricuspid annuloplasty.

### Early postoperative outcomes

Table 4 shows early postoperative outcomes of the participants. One patient in the non-BPA group died in-hospital of cardiopulmonary failure. The BPA group had a significantly lower incidence of the composite event than the non-BPA group (4.8% vs 35.1%, *P* = 0.011). Although the univariable analysis revealed that the non-BPA group and mPAP were significantly associated with the composite event, the multivariable analysis did not reveal any statistically significant factors (Table 5).

**Table 4:** Early postoperative outcomes

Variable	BPA group	non-BPA group	*P*-value
Complications			
In-hospital deaths	0	2.7 (1)	1.000
Rescue BPA, % (n)	0	2.7 (1)	1.000
Prolonged ventilation (> 3 days)	4.8 (1)	24.3 (9)	0.077
Tracheotomy, % (n)	4.8 (1)	10.8 (4)	0.644
Deep sternal wound infection, % (n)	0	2.7 (1)	1.000
Cerebral infarction, % (n)	0	2.7 (1)	1.000
Increased serum creatinine (> 0.5 mg/dl), % (n)	0	0	1.000
Prolonged ICU stay (> 10 days), % (n)	4.8 (1)	24.3 (9)	0.077
Composite event^a^, % (n)	4.8 (1)	35.1 (13)	0.011
Total number of complications including mechanical support requirement after PEA, n	5	35	n/a
Duration of hospital stay, days	30 [25, 36]	43 [32, 59]	0.006

Data are presented as medians [interquartile range].

a A composite event comprises at least 1 of the following complications: postoperative extracorporeal membrane oxygenation, postoperative intra-aortic balloon pump use, in-hospital death, rescue pulmonary balloon plasty, prolonged ventilation, tracheostomy, deep sternal wound infection, cerebral infarction and prolonged stay in the intensive care unit.

BPA: balloon pulmonary angioplasty; ICU: intensive care unit; PEA: pulmonary endarterectomy

**Table 5:** Univariable and multivariable logistic regression models for evaluating the associated factors for the composite event

	Univariable analysis			Multivariable analysis		
Variable	Odds ratio	95% CI	*P*-value	Odds ratio	95% CI	*P*-value
non-BPA group (vs BPA group)	10.83	1.30, 90.14	0.028	7.52	0.86, 65.49	0.068
Age (years)	0.98	0.93, 1.03	0.386			
Female	1.03	0.29, 3.60	0.965			
NYHA/WHO functional class III, IV (vs II)	1.90	0.46, 7.85	0.377			
mPAP (mmHg)	1.14	1.00, 1.29	0.047	1.10	0.95, 1.26	0.191
Current smoker	0.49	0.05, 4.44	0.523			
Former smoker	0.65	0.16, 2.72	0.556			
Thrombotic predisposition	1.23	0.28, 5.44	0.787			
Hypertension	1.59	0.47, 5.33	0.454			
Dyslipidaemia	0.75	0.14, 4.03	0.737			
History of symptomatic cerebral infarction	5.73	0.85, 38.61	0.073			
Without oral PAH-targeted medical treatment	0.49	0.05, 4.44	0.523			
Surgical classification III, IV (vs I, II)	3.73	0.66, 21.09	0.137			
Concomitant surgery	2.16	0.58, 8.05	0.251			

Concomitant surgery comprises patients with tricuspid annuloplasty, the maze procedure, coronary artery bypass grafting, mitral valve repair, surgical aortic valve replacement or ascending aortic replacement.

BPA: balloon pulmonary angioplasty; CI: confidence interval; mPAP: mean pulmonary arterial pressure; NYHA/WHO: New York Heart Association/World Health Organization; PAH: pulmonary arterial hypertension.

### Right heart catheterization after pulmonary endarterectomy

We found that mPAP and PVR significantly decreased after PEA in both groups (Fig. 2).

### Survival

**Figure 3:**
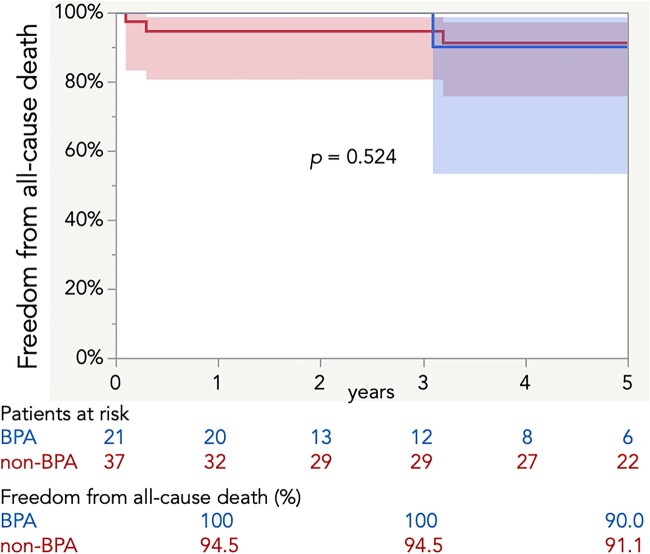
Survival rates with 95% confidence intervals. BPA: balloon arterial pressure.

The median follow-up period was shorter in the BPA group than in the non-BPA group (3.0 vs 5.8 years, *P* = 0.021) (Supplementary Table S1). In addition to the in-hospital death, 2 patients died of a cerebral haemorrhage and lung cancer, respectively, in the BPA group, and 3 patients died of cardiac issues, unknown aetiology and malignancy, respectively, in the non-BPA group. The Kaplan–Meier method revealed no significant difference in survival rates between the 2 groups (Fig. 3).

## DISCUSSION

The present study suggested that having BPA before PEA was associated with a decrease in mPAP and PVR as well as an increase in cardiac output before PEA. Furthermore, compared with direct PEA, the combination of performing BPA before PEA was associated with a lower incidence of the early postoperative composite event in high-surgical-risk patients with CTEPH.

### Surgical risk reduction from performing balloon pulmonary angioplasty before pulmonary endarterectomy

Right ventricular (RV) function acutely decreases during on-pump cardiac surgery, and the decrease continues for some time after the operation [11]. However, the reduced RV function may be well tolerated by most patients undergoing such operations because RV afterload typically improves after cardiac surgery. In contrast, patients with pre-existing RV dysfunction may not endure a cardiac operation, particularly those with severe PA occlusive disease or combined cardiac operations due to increased cardiac damage. Such high-surgical-risk patients are likely to develop postoperative complications related to respiratory and/or cardiac failure. The results of this study revealed that patients who underwent direct PEA had a greater incidence of early postoperative complications, mainly including mechanical circulatory and ventilatory support-related complications, compared with those who underwent the combination therapy. Because BPA has the beneficial effect of improving right heart function following PA pressure reduction and a subsequent increase in cardiac output [12], BPA carried out before PEA may reduce complications after PEA in high-surgical-risk patients with CTEPH.

Although BPA has emerged as a therapeutic option for patients with CTEPH who are considered inoperable or who refuse PEA [13], its role in the treatment algorithm has been re-evaluated for patients with residual or recurrent PAH [2, 3]. It is reasonable to consider additional BPA after PEA for such patients, and several studies have demonstrated improved mPAP, residual symptoms and exercise capacity after BPA [5–7]. Conversely, the efficacy of performing BPA first, followed by PEA, is unknown, with only a few reports with small numbers of patients being published [14–16]. This situation may be attributed to the fact that the plane of the PEA dissection may deteriorate or become distorted during BPA, resulting in an impossible-to-correct dissection and in vascular injuries during PEA. However, the present study did not identify any patient in whom adequate plane dissection was difficult or patients with vascular injuries, although PEA was performed for most lesions that were first treated with BPA. Two recent case reports of patients with high PVR (985 and 1569 dyn·s/cm^5^, respectively) demonstrated reduced preoperative PVR when BPA preceded PEA for both lungs, and PEA after BPA resulted in successful outcomes [14, 15]. Conversely, performing BPA for 1 lung (including surgically inaccessible lesions) followed by PEA for the other lung (including proximal lesions) is an alternative strategy. Mercier *et al.*[16] reported the outcomes of this strategy in 13 of 418 patients who underwent PEA for CTEPH. Notably, these 13 patients were older than those who underwent PEA alone (72 vs 60 years, *P* = 0.014). Compared with the use of PEA alone, performing BPA first followed by PEA resulted in a lower 90-day death rate, shorter cardiopulmonary bypass time and a similar percentage decrease in mPAP after PEA.

Mechanical vascular injuries, including wire perforation, balloon over-dilatation and high-pressure injection of contrast media, are considered to be the major causes of BPA-related complications [17, 18]. Complications related to performing BPA first, followed by PEA, may aggravate a patient’s condition and further complicate the operation. Although 4 patients in the present study experienced minor complications requiring NPPV, no adverse effects of these complications on the operation were observed. However, several patients undergoing BPA may experience major complications, including death or the need for intubation or ECMO [13]. In contrast to BPA in inoperable patients, which is done to relieve PAH and other symptoms, the treatment goal of performing BPA first, followed by PEA, is to reduce the operative risk. Obtaining effective results, such as a minimal number of sessions and complications, when performing BPA followed by PEA is required to help prevent complications after PEA and allow adequate time for surgical preparation.

### High-surgical-risk patients with chronic thromboembolic pulmonary hypertension

Patients with severely deteriorated pulmonary haemodynamics or significant comorbidities may not receive the full benefit of PEA despite the fact that the CTEPH cases are technically operable. Madani *et al.* (19) reported that a preoperative PVR of >1000 dyn·s/cm^5^ was associated with a 3–4 times higher risk of postoperative death, and Ishida *et al.* (20) demonstrated a preoperative PVR of >1052 dyn·s/cm^5^ as a risk factor for in-hospital death. The postoperative mortality rate was reported to increase as the PVR increased (21), and Saouti *et al.* (22) reported preoperative PVR of 645 dyn·s/cm^5^ and high mPAP of >46 mmHg as the cut-off values for distinguishing between high- and low-risk cases for postoperative deaths. In addition, residual PAH, postoperative PVR of >425–500 dyn·s/cm^5^ or mPAP of >34–38 mmHg, was reported to be related with postoperative deaths [19, 20, 23]. Based on these outcomes, severe PAH with a PVR of ≥1000 dyn·s/cm^5^ or an mPAP of ≥45 mmHg was defined as the first criterion to identify patients at high surgical risk in the present study. Among patients with characteristics other than those mentioned in the first criterion, patients with moderate PAH with an mPAP of >38 mmHg and comorbidities, including advanced age, obesity, renal dysfunction or the need for concomitant cardiac surgery, which may affect postcardiac surgery outcomes [24, 25], were also defined as high-surgical-risk patients for PEA.

### Pulmonary endarterectomy in the balloon pulmonary angioplasty era

Maintaining stable preoperative haemodynamics with lower mPAP and PVR should be more comfortable for all surgeons who perform PEA. The results of this study suggested that a CTEPH team can stabilize the haemodynamics of patients with CTEPH by performing BPA with or without MT before PEA. Durable surgery without major complications is important for high-surgical-risk patients, because postoperative alternative therapy, including additional BPA and/or MT, is often available even when there is residual PAH [3, 5–7]. BPA before or after PEA would lead to a better quality of life for patients.

### Study limitations

This study has some limitations. First, it was a single-centre retrospective observational study with a small number of patients. In addition, performing BPA first followed by PEA was initiated in 2014, and >50% of patients at high surgical risk did not have an opportunity to undergo this procedure before that date. Second, various MTs were attempted preoperatively in most patients, but their additional effects were not evaluated. Third, the effects of the number of sessions in which BPA before PEA and the intervals from BPA to PEA were not assessed. Fourth, long-term outcomes, effects of additional MT or BPA after PEA and data from functional capacity assessment, including 6-min walk tests and other physical capacity assessments, were not evaluated. Fifth, the non-BPA group included a larger number of early-period cases than the BPA group. Because the surgical technique and primary surgeon did not change throughout the study period, these factors have little impact on postoperative outcomes. Finally, although multivariable analysis revealed no associated factors for the early postoperative composite event, several factors, including age and concomitant operations, may have influenced the outcome. Nevertheless, multicentre registries or randomized trials are warranted to address these issues.

## CONCLUSIONS

The combination therapy of BPA preceding PEA and PEA can be a feasible and effective risk-reduction strategy compared with direct PEA in high-surgical-risk patients with CTEPH.

## Supplementary Material

ivad031_Supplementary_Data

## Data Availability

The article’s data will be shared on reasonable request to the corresponding author. **Yusuke Shimahara:** Conceptualization, Data curation, Formal analysis, Investigation, Writing-original draft; **Shun Suzuki:** Data curation, Investigation; **Toshiki Fujiyoshi:** Investigation; **Sayaka Honda:** Data curation; **Nobusato Koizumi:** Investigation; **Jun Yamashita:** Investigation; **Yuichi Sasaki:** Investigation; **Ryosuke Ito:** Investigation; **Lisa Takahashi:** Investigation; **Michikazu Nakai:** Formal analysis; **Hitoshi Ogino:** Investigation: Supervision, Writing-review & editing.

## Abbreviations

BPA: Balloon pulmonary angioplasty
CTEPH: Chronic thromboembolic pulmonary hypertension
ECMO: Extracorporeal membrane oxygenation
IABP: Intra-aortic balloon pump
mPAP: Mean pulmonary arterial pressure
MT: Medical treatment
NPPV: Noninvasive positive pressure ventilation
PA: Pulmonary artery
PEA: Pulmonary endarterectomy
PAH: Pulmonary arterial hypertension
PVR: Pulmonary vascular resistance
RV: Right ventricular
