# Supplementary material for: Balloon pulmonary angioplasty followed by pulmonary endarterectomy: Combination treatment for high-surgical-risk patients with chronic thromboembolic pulmonary hypertension
Source: Interdiscip Cardiovasc Thorac Surg. 2023 Feb 24;36(3):ivad031. doi: 10.1093/icvts/ivad031 (PMC9985147; doi:10.1093/icvts/ivad031)
Supplement: ivad031_Supplementary_Data [file ivad031_supplementary_data.zip › Supplementary Table S1_2nd version.docx]

Supplementary Table S1. Mortality during follow-up period

| Variable | BPA group | non-BPA group | P-value |
| --- | --- | --- | --- |
| Follow-up period, median years | 3.0 [1.3, 5.1] | 5.8 [2.3, 7.1] | 0.021 |
| Follow-up rate, % (n) | 100 (21) | 91.9 (34) | 0.547 |
| All-cause death, % (n) | 9.5 (2) | 10.8 (4) | 1.000 |
| Cardiac cause, % (n) | 0 | 5.4 (2) | 0.530 |
| Unknown cause, % (n) | 0 | 2.7 (1) | 1.000 |
| Malignancy, % (n) | 4.8 (1) | 2.7 (1) | 1.000 |
| Cerebral bleeding, % (n) | 4.8 (1) | 0 | 0.362 |

BPA, balloon pulmonary angioplasty

Data are presented as medians [interquartile range].
