# Supplementary figures and images for: Balloon pulmonary angioplasty followed by pulmonary endarterectomy: Combination treatment for high-surgical-risk patients with chronic thromboembolic pulmonary hypertension
Source: Interdiscip Cardiovasc Thorac Surg. 2023 Feb 24;36(3):ivad031. doi: 10.1093/icvts/ivad031 (PMC9985147; doi:10.1093/icvts/ivad031)

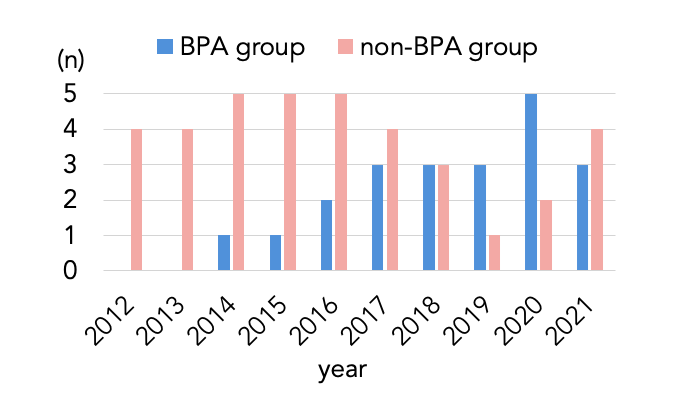

Supplement: ivad031_Supplementary_Data [file ivad031_supplementary_data.zip › Supplementary Figure S1_2nd version.tif]
